# Identification of common immunodominant antigens of *Eimeria tenella*, *Eimeria acervulina* and *Eimeria maxima* by immunoproteomic analysis

**DOI:** 10.18632/oncotarget.16824

**Published:** 2017-04-04

**Authors:** Lianrui Liu, Xinmei Huang, Jianhua Liu, Wenyu Li, Yihong Ji, Di Tian, Lu Tian, Xinchao Yang, Lixin Xu, Ruofeng Yan, Xiangrui Li, Xiaokai Song

**Affiliations:** ^1^ College of Veterinary Medicine, Nanjing Agricultural University, Nanjing 210095, China; ^2^ Institute of Veterinary Medicine, Jiangsu Academy of Agricultural Science, Nanjing 210014, China

**Keywords:** Eimeria, sporozoites, common immunodominant antigens, immunoproteomics

## Abstract

Clinical chicken coccidiosis is mostly caused by simultaneous infection of several *Eimeria* species, and host immunity against *Eimeria* is species-specific. It is urgent to identify common immunodominant antigen of *Eimeria* for developing multivalent anticoccidial vaccines. In this study, sporozoite proteins of *Eimeria tenella*, *Eimeria acervulina* and *Eimeria maxima* were analyzed by two-dimensional electrophoresis (2DE). Western bot analysis was performed on the yielded 2DE gel using antisera of *E. tenella E. acervulina* and *E. maxima* respectively. Next, the detected immunodominant spots were identified by comparing the data from MALDI-TOF-MS/MS with available databases. Finally, *Eimeria* common antigens were identified by comparing amino acid sequence between the three *Eimeria* species. The results showed that analysis by 2DE of sporozoite proteins detected 629, 626 and 632 protein spots from *E. tenella*, *E. acervulina* and *E. maxima* respectively. Western bot analysis revealed 50 (*E. tenella*), 64 (*E. acervulina*) and 57 (*E. maxima*) immunodominant spots from the sporozoite 2DE gels of the three *Eimeria* species. The immunodominant spots were identified as 33, 27 and 25 immunodominant antigens of *E. tenella, E. acervulina* and *E. maxima* respectively. Fifty-four immunodominant proteins were identified as 18 ortholog proteins among the three *Eimeria* species. Finally, 5 of the 18 ortholog proteins were identified as common immunodominant antigens including elongation factor 2 (EF-2), 14-3-3 protein, ubiquitin-conjugating enzyme domain-containing protein (UCE) and glyceraldehyde-3-phosphate dehydrogenase (GAPDH). In conclusion, our results not only provide *Eimeria* sporozoite immunodominant antigen map and additional immunodominant antigens, but also common immunodominant antigens for developing multivalent anticoccidial vaccines.

## INTRODUCTION

Avian coccidiosis, a major parasitic disease of chickens worldwide, was caused by intestinal infection of *Eimeria* spp. [1]. It causes reduction in weight gain and poor feed-conversion, and death of the chickens, leading to an estimated annual economic loss of more than US$3 billion to the global poultry [2, 3]. The species of *E. tenella*, *E. acervulina* and *E. maxima* are the most important in terms of global disease burden and economic impact [2, 4].

Present control strategy against this disease relies on anticoccidial drugs and live vaccines containing virulent or attenuated strains of *Eimeria* [5]. However, chemical residues, emergence of drug-resistant parasites and the high cost associated with the development of new drugs results in serious problems. Moreover, the live vaccines have inherent production limitations, risk of vaccinal pathogenicity as well as the potential reversion to a pathogenic form, and cost issues [2, 6, 7]. Thus, new vaccines containing either defined immunodominant antigens or based on recombinant DNA technology have been or are being developed [2, 8, 9]. Clinical coccidiosis is mainly caused by co-infection with multiple species of *Eimeria* [10, 11], hence, a practical novel anticoccidial vaccine should contain the common antigens among *Eimeria* or antigens from multiple *Eimeria* species. Therefore, exploring immunodominant antigens, especially common antigens of *Eimeria*, is essential for developing novel vaccine against the simultaneous infection clinically.

Here, we described immunoproteomic analysis of *Eimeria tenella*, *Eimeria acervulina* and *Eimeria maxima*. A batch of immunodominant antigens was identified, with 33, 27 and 25 found in *E. tenella*, *E. acervulina* and *E. maxima*, respectively. Eighteen ortholog proteins and 5 common immunodominant antigens across the three *Eimeria* species were identified. Our results provide additional immunodominant antigens and common antigens for the development of multivalent vaccines against *Eimeria*.

## RESULTS

### Sporozoite 2DE gel profile of *E. tenella, E. acervulina* and *E. maxima*

**Figure 1:**
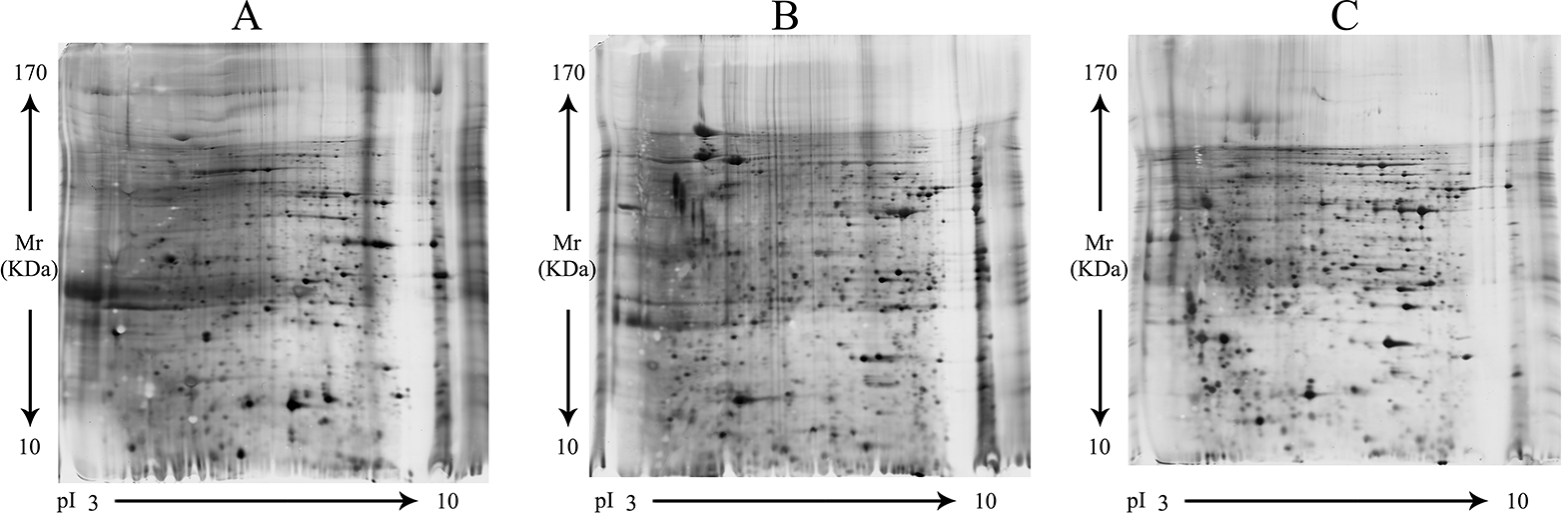
Sporozoite 2-DE gel profile of *E. tenella, E. acervulina* and *E. maxima* (**A**) *E. tenella*, (**B**) *E. acervulina*, (**C**) *E. maxima*. Soluble proteins (400 μg) from sporozoite of the three species were resolved by IEF over a broad, non-linear pH 3, 10 range followed by molecular mass on a 12.5% w/v acrylamide gel under denaturing conditions. Protein spots are visualized using silver stain.

The separation by 2-DE of 400 μg solubilized sporozoite proteins detected 629, 626 and 632 spots of *E. tenella, E. acervulina* and *E. maxima*, respectively. Most spots were located between 13 and 140 kDa (Figure 1). Analysis with ImageMaster 2D Platinum (Version 5.0, GE Amersham) revealed 22 spots shared among all these species.

### Detection of immunodominant spots by Western blot

**Figure 2:**
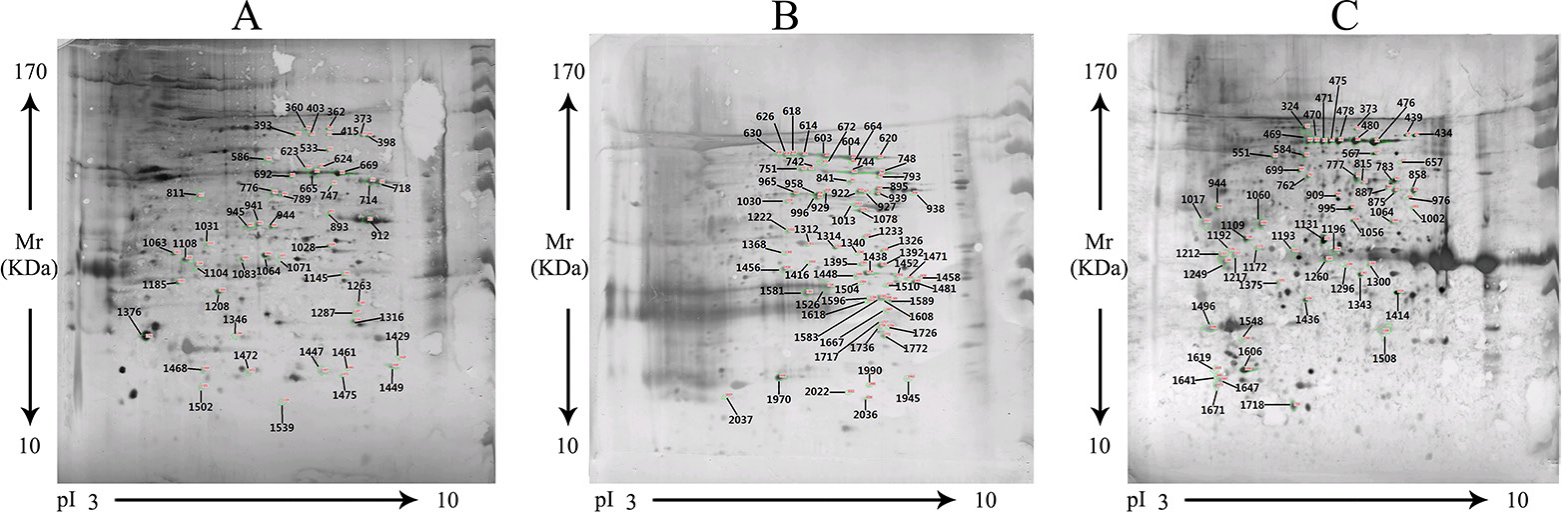
Western blot analysis of the sporozoite 2DE gels of *E. tenella, E. acervulina* and *E. maxima* with anti-*E. tenella, anti-E. acervulina* and anti-*E. maxima* sera (**A**) *E. tenella*, (**B**) *E. acervulina*, (**C**) *E. maxima*.

**Figure 3:**
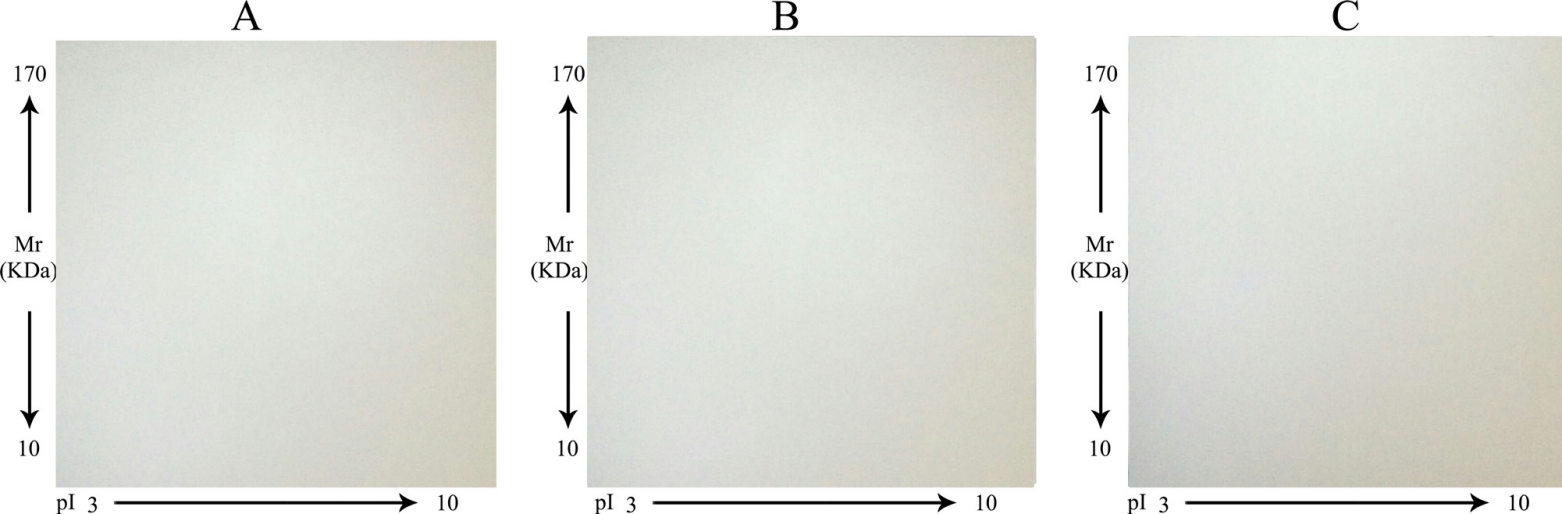
Western blot analysis of the sporozoite 2DE gels of *E. tenella*, *E. acervulina* and *E. maxima* with sera from negative control chickens (**A**) *E. tenella*, (**B**) *E. acervulina*, (**C**) *E. maxima*.

Sporozoite 2DE gels of *E. tenella*, *E. acervulina* and *E. maxima* were analyzed by western blot using the corresponding antisera of these *Eimeria* species separately. Western blot profiles of the 2DE gel were shown in Figure 2. Immunodominant spots were observed on the western blot profiles of the three *Eimeria* species. Comparison with ImageMaster 2D Platinum revealed that 50 (*E. tenella*), 64 (*E. acervulina*) and 57 (*E. maxima*) immunodominant spots had high similarity between the 2DE gel profile and western blot profile. When the same western blot was probed with sera from negative control chickens, no proteins were detected (Figure 3).

### Immunodominant proteins analysis and identification using NCBI and Uniport database

All the immunodominant spots (171) detected by western blot were analyzed by MALDI-TOF-MS/MS. The obtained peptide mass fingerprint dates were submitted to MASCOT Sequence Query server (http://www.matrixscience.com) for identification against nonredundant NCBI database (http://www.ncbi.nlm.nih.gov/BLAST) and the uniprot database (http://www.uniprot.org/). Identification required a MASCOT confidence interval of 95%. As shown in Table 1, 112 spots were identified in the databases as corresponding to 85 *Eimeria* proteins, including 33 of *E. tenella*, 27 of *E. acervulina* and 25 of *E. maxima*. Fifty-four out of the 85 immunodominant proteins were 18 kinds of ortholog proteins among the three *Eimeria* species. Table 2 showed amino acids similarity of the 18 ortholog proteins between the three *Eimeria* species. All the ortholog proteins shared sequence similarity of more than 63% between the three *Eimeria* species except peroxiredoxin. Five of the ortholog proteins even shared sequence similarity of more than 93% between the three *Eimeria* species, namely, elongation factor 2 (EF-2), 14-3-3 protein, ubiquitin-conjugating enzyme domain-containing protein (UCE), glyceraldehyde-3-phosphate dehydrogenase (GAPDH) and transhydrogenase. Therefore, the five proteins were identified as common immunodominant antigens among the three *Eimeria* species. Since there were no matched proteins in the database, 59 spots were not identified successfully.

**Table 1:** Identification of *E. tenella>, E. acervulina* and *E. maxima* sporozoite proteins in NCBI and Uniprot database using data from MALDI-TOF-MS/MS analyses

Spot ID^a^	Identified protein	Database ID
A929	14 kDa phosphohistidine phosphatase, putative (*E. acervulina*)	CDI76382.1
M1436	14 kDa phosphohistidine phosphatase, putative (*E. maxima*)	CDJ61236.1
T1468, T669	14 kDa phosphohistidine phosphatase, putative (*E. tenella*)	CDJ40270.1, U6KXP8|U6KXP8
T1475	14-3-3 protein (*E. tenella*)	gi|21541950
A1222, A1392, A1395	14-3-3 protein, putative (*E. acervulina*)	XP_013250285
M567	14-3-3 protein, putative (*E. maxima*)	XP_013335589
M1056, M1064, M1414	56 kDa gametocyte antigen (*E. maxima*)	gi|25989587
T1064	56 kDa gametocyte antigen, related (*E. tenella*)	CDJ41536.1
A620	56 kDa gametocyte antigen, related OS= *E. acervulina*	CDI78697.1
M1131	82 kDa gametocyte antigen OS= *E. maxima*	Q86LH7|Q86LH7
T1472	Actin depolymerizing factor (*E. tenella*)	ABM89551.1
A1510	Actin depolymerizing factor, putative (*E. acervulina*)	CDI83856.1
M1647, M1343	Actin depolymerizing factor, putative (*E. maxima*)	CDJ60866.1
M469	Actin, putative OS=*E. maxima*	U6M655|U6M655_
T941	Alanine dehydrogenase, putative (*E. tenella*)	CDJ43978.1
A604	Alanine dehydrogenase, putative OS=*E. acervulina*	U6GM75|U6GM75
A1448	Aldo/keto reductase family oxidoreductase, putative (*E. acervulina*)	CDI80286.1
T1145	Aldo/keto reductase family oxidoreductase, putative (*E. tenella*)	CDJ45355.1
M584	Aldo/keto reductase family oxidoreductase, putative OS=*E. maxima*	CDJ57423.1
T393	Aspartyl proteinase (Eimepsin) OS=*E. tenella*	Q9GN67|Q9GN67
A1458	Cytosol aminopeptidase, putative (*E. acervulina*)	CDI77668.1
T362	Cytosol aminopeptidase, putative OS=*E. tenella*	U6KUY7|U6KUY7
M1619, M1641	Dihydrolipoyl dehydrogenase OS=*E. maxima*	CDJ55972.1
A1368	Dihydrolipoyl dehydrogenase, putative (*E. acervulina*)	CDI82910.1
T1376	Dihydrolipoyl dehydrogenase, putative (*E. tenella*)	CDJ38931.1
T415	Dynein heavy chain protein, related OS=*E. tenella*	U6KWQ6|U6KWQ
A1970	Elongation factor 1-alpha OS=*E. acervulina*	U6GWZ2|U6GWZ
M480, M470	Elongation factor 1-alpha, putative (*E. maxima*)	gi|557198794
M478	Elongation factor 2, putative (*E. maxima*)	gi|557157066
T811	Elongation factor 2, putative (*E. tenella*)	gi|557140071
A939, A965	Elongation factor 2, putative OS=*E. acervulina*	U6GRG2|U6GRG2
T747	Enolase 2, putative (*E. tenella*)	CDJ38513.1
A1456	Enolase 2, putative OS=*E. acervulina*	CDI82390.1
M1548	Enolase 2, putative OS=*E. maxima*	CDJ56218.1
A744, A742	Fructose-bisphosphate aldolase OS=*E. acervulina*	CDI80998.1
T398	Fructose-bisphosphate aldolase OS=*E. tenella*	CDJ43237.1
M1060	Fructose-bisphosphate aldolase, related (*E. maxima*)	CDJ59017.1
A751, A748	Glyceraldehyde-3-phosphate dehydrogenase OS=*E. acervulina*	CDI78463.1
T403	Glyceraldehyde-3-phosphate dehydrogenase OS=*E. tenella*	CDJ43289.1
M1249	Glyceraldehyde-3-phosphate dehydrogenase, putative (*E. maxima*)	CDJ57266.1
A1581, A1340	Haloacid dehalogenase-like hydrolase domain-containing protein, putative (*E. acervulina*)	CDI76904.1
T1287	Haloacid dehalogenase-like hydrolase domain-containing protein, putative (*E. tenella*)	CDJ37571.1
M471	Haloacid dehalogenase-like hydrolase OS=E. maxima	CDJ61159.1
T1185	Hypothetical protein (*E. tenella*)	gi|357017711
T1316	Hypothetical protein (*E. tenella*)	AET50460.1
T1447	Hypothetical protein (*E. tenella*)	AET50635.1
T1208	Hypothetical protein (*E. tenella*)	gi|357017711
A841	Hypothetical protein, conserved (*E. acervulina*)	CDI78255.1
T1263	Hypothetical protein, conserved (*E. tenella*)	CDJ37254.1
M1375	KH domain-containing protein, putative (*E. maxima*)	CDJ59518.1
T776	KH domain-containing protein, putative (*E. tenella*)	CDJ38027.1
A895	KH domain-containing protein, putative OS=*E. acervulina*	CDI84033.1
A1618	Lactate dehydrogenase (*E. acervulina*)	ACM77785.1
M783, M762	Lactate dehydrogenase OS=*E. maxima* GN=LDH	Q8I8U3|Q8I8U3_E
T718	Lactate dehydrogenase OS=*E. tenella*	CDJ37067.1
A1736	Microneme 2 (*E. acervulina*)	KR063282.1
M1002	Microneme protein 7 OS=*E. maxima* GN=mic7	G0LEU8|G0LEU8
T1502	Microneme protein MIC3, partial (*E. tenella*)	gi|40549149
T1449	Mitochondrial branched-chain alpha-keto acid dehydrogenase E1, putative (*E. tenella*)	CDJ43491.1
A626	Mitochondrial branched-chain alpha-keto acid dehydrogenase E1, putative, partial (*E. acervulina*)	CDI81447.1
T692	Nucleoside diphosphate kinase OS=*E. tenella*	U6KIW7|U6KIW7
M373	Nucleoside diphosphate kinase, putative (*E. maxima*)	CDJ59193.1
A927	Peroxiredoxin, putative (*E. acervulina*)	CDI84011.1
M1109	Peroxiredoxin, putative OS=*E. maxima*	CDJ59087.1
T360	Peroxisomal catalase, putative OS=*E. tenella*	CDJ43752.1
A1233, A922	Proteasome subunit alpha type 7, putative (*E. acervulina*)	CDI79975.1, U6GIG8|U6GIG8_
T893, T624	Proteasome subunit alpha type 7, putative (*E. tenella*)	CDJ41908, U6KYA5|U6KYA5
M475, M476, M434, M875	Purine nucleoside phosphorylase, putative (*E. maxima*)	CDJ57289.1
T1083	Purine nucleoside phosphorylase, putative (*E. tenella*)	CDJ44020.1
A996	Purine nucleoside phosphorylase, putative OS=*E. acervulina*	CDI78710.1
T533	Putative uncharacterized protein OS=*E. tenella*	H9B9×1|H9B9×1_
A603, A614, A618	Pyruvate kinase, putative (*E. acervulina*)	CDI78351.1
A1326	Sporozoite antigen, partial (*E. acervulina*)	CAA33905.1
T623	Transhydrogenase (Fragment) OS=*E. tenella*	Q24937|Q24937
T714	Transhydrogenase OS=*E. tenella* GN=7B2 PE=4	Q07600|Q07600
A1078, A1717	Transhydrogenase, putative (*E. acervulina*)	CDI76761.1
M1718	Transhydrogenase, putative (*E. maxima*)	CDJ61620.1
T1028, T665	Triosephosphate isomerase (*E. tenella*)	CDJ37485.1, H9BA04|H9BA04
A958, A1772	Triosephosphate isomerase OS=*E. acervulina*	U6GH90|U6GH90, CDI79606.1
M1606, M1212	Triosephosphate isomerase, putative (*E. maxima*)	CDJ60494.1
A1481, A1438, A1452	Ubiquitin-conjugating enzyme domain-containing protein, putative (*E. acervulina*)	gi|557117367
M699	Ubiquitin-conjugating enzyme domain-containing protein, putative (*E. maxima*)	CDJ61561
T1063	Ubiquitin-conjugating enzyme domain-containing protein, putative (*E. tenella*)	XP_013236351
M995, M909	Uncharacterized protein OS=*E. maxima*	U6M744|U6M744_
M858	Uncharacterized protein OS=*E. maxima*	U6LYQ2|U6LYQ2

Note: 1.^a^ Number of the spot in the 2-DE gel and the western blot membrane; 2. A: *E. acervulina*, T: *E. tenella*, M: *E. maxima*.

**Table 2:** Amino acid sequence similarity of the 18 immunodominant ortholog proteins among *E. tenella E. acervulina and E. acervulina*

Immunodominant ortholog proteins	Amino acid sequence similarity between		
	*E. tenella* and *E. acervulina*	*E. acervulina* and *E. maxima*	*E. tenella* and *E. maxima*
Elongation factor 2, putative	99%	99%	99%
14-3-3 protein	97.5%	99.6%	97.1%
Ubiquitin-conjugating enzyme domain-containing protein, putative	96.6%	97.4%	98.3%
Glyceraldehyde-3-phosphate dehydrogenase	94.1%	95.9%	92.6%
Transhydrogenase	93.4%	94.5%	92.9%
Actin depolymerizing factor	89.8%	93.2%	88.1%
Triosephosphate isomerase	82.1%	92.0%	78.9%
Fructose-bisphosphate aldolase	77.8%	99.6%	80.4%
Purine nucleoside phosphorylase, putative	79.3%	81.2%	81.2%
Aldo/keto reductase family oxidoreductase, putative	78.5%	79.3%	85.7%
Dihydrolipoyl dehydrogenase	75.7%	81.0%	84.1%
Enolase 2	76.4%	86.2%	72.7%
Haloacid dehalogenase-like hydrolase domain-containing protein	74.5%	82.0%	76.2%
KH domain-containing protein, putative	71.5%	77.9%	69.3%
Lactate dehydrogenase	68.2%	80.6%	63.6%
14 kDa phosphohistidine phosphatase, putative	64.0%	76.5%	64.2%
56 kDa gametocyte antigen	68.0%	77.3%	65.9%
Peroxiredoxin	13.8%	89.3%	16.2%

## DISCUSSION

The immunity elicited by infections with *Eimeria* is species specific and an effective recombinant vaccine should include common protective antigens among several *Eimeria* species [12–14]. Some researchers have reported several *Eimeria* common antigens. Talebi reported a conserved immunodominant protein band (45 kDa) among sporulated oocysts of five *Eimeria* species (*E. acervulina, E. maxima, E. necatrix, E. praecox* and *E. tenella*) recognized by chicken anti-*E. maxima* serum. Sasai and colleagues reported a common antigen present on the apical complex of all chicken *Eimeria* sporozoites [15]. Constantinoiui and colleagues reported a highly conserved apical antigen among *Eimeria* species [13]. However, the reported common antigens are not specific. In the present study, we identified at least 5 specific *Eimeria* common immunodominant antigens by immunoproteomic analysis. Our research provided additional candidate common antigens for developing multivalent vaccines against simultaneous infection by several *Eimeria* species.

Since antibodies could confer protective immunity against *Eimeria* [17–20], in addition, the induced antibodies are relatively long lasting and easy for collection [13, 17, 21]. Therefore, in some previous reports, *Eimeria* antisera were used to identify immunodominant *Eimeria* antigens. For example, Réfega et al. obtained a total of 119 cDNA clones by immunoscreening *E. tenella* libraries using intestinal antibodies [22]. Laurent et al. screened a 19-kilodalton antigen present in several *Eimeria* species using sera raised to *E. acervulina* or *E. tenella* [23]. Some immunodominant antigens were screened from cDNA libraries by corresponding *Eimeria* antisera, such as TA4 [24], LPMC-61 [25], rhomboid proteins ETRH01 of *E. tenella* [26] and so on, and these identified immunodominant antigen were further demonstrated to be able to confer protection against *Eimeria* challenge [27, 28]. Therefore, we also used the *Eimeria* antisera to screen the immunodominant antigens of *Eimeria* species and obtained 85 immunodominant proteins. Part of the identified antigens were also identified as immunodominant antigens in previous studies, including MIC3, pyruvate kinase, enolase, actin, aspartyl proteinase, 14-3-3 protein, lactate dehydrogenase and so on [29–31]. Some of the identified antigens have been demonstrated to be able to confer protection against *Eimeria* challenge, such as lactate dehydrogenase [32], microneme 2 [33], microneme 7 [34] and so on. Therefore, the immunodominant antigens identified in this study have the potential for conferring protection against *Eimeria* challenge.

In this study, most of the identified immunodominant antigens shared orthologous relationships across the three *Eimeria* species. Nearly all the ortholog proteins shared amid acid sequence similarity of more than 63%, furthermore, five proteins shared sequence similarity of more than 93% and were identified as common immunodominant proteins among the three *Eimeria* species. In addition, we compared the available amid acid sequence of the five identified common immunodominant proteins among 7 chicken *Eimeria* species, and found that nearly all the sequence similarity among the 7 *Eimeria* species were more than 90% except *E. mitis* UCE, indicating the 5 protein were highly conserved among 7 chicken *Eimeria*. Our further studies demonstrated the identified common antigen 14-3-3 protein and GAPDH could confer effective protection against challenge by several *Eimeria* species (unpublished data). Taken together, our data demonstrated that immunoproteomics screening could be an efficient approach for identifying common immunodominant proteins of *Eimeria* species.

Biological functions of the five common antigens have been generally described in some protozoa. Eukaryotic elongation factor 2 plays crucial role in the elongation stage of mRNA translation in eukaryotes, by mediating the translocation of the ribosome relative to the mRNA after addition of each amino acid to the nascent chain [35, 36]. The 14-3-3 proteins are a family of conserved regulatory molecules expressed in all eukaryotic cells. And play important roles in extensively regulatory processes, such as mitogenic signal transduction, apoptotic cell death, cell cycle control, and protein localization [37, 38]. *E. tenella* 14-3-3 protein could interact with the telomerase RNA-binding domain of telomerase reverse transcriptase [39]. UCE is a member of the family of ubiquitin-conjugating (E2) enzymes characterized by the presence of a highly conserved ubiquitin-conjugating (UBC) domain. E2 enzymes are well-conserved in eukaryotes and involved in Ub/UBL-modification pathways, and play central roles in processes like regulating protein degradation, function, and localization, thereby controlling the biology of the eukaryotic cell [40]. GAPDH is a key glycolytic enzyme in the process of metabolism of coccidian, as several pathogenic protozoa entirely depend on glycolysis as the source of ATP in the host [41, 42]. Transhydrogenase catalyses transhydrogenation between analogues of NAD(H) and NADP(H). A transhydrogenase was found to be located in the *Eimeria* refractile body and might function in relation with the ATP hydrolysis and respiration in the process of oocysts sporulation [43]. However, their specific biological functions in *Eimeria* need further studies.

In previous studies, the similarities of conserved or common antigens ranged from 70% to 99% [44–46]. In theory, the higher similarity a protein among several species is, the more conserved the protein is. Thus, we used a high threshold of 93% to define the common antigens. Certainly, we can use a lower threshold less than 93%. If so, more antigens would be identified as common antigens.

It has been reported that some of the five common immunodominant antigens were protective in protozoa and other parasites. *Toxoplasma gondii* 14-3-3 protein was proved to be a potential vaccine candidate against toxoplasmosis [47]. *Leishmania* elongation factor 2 was identified as T cell-stimulating antigen and might constitute potential vaccine candidates for leishmaniasis [48]. Elongation factor 1-Alpha was reported as protective antigen in *Toxoplasma gondii* and *Cryptosporidium parvum* [49, 50]. GAPDH was proved to confer protection against *Haemonchus contortus* and *Schistosoma mansoni* [51, 52]. Protozoal GAPDHs were suggested as a potential antiparasitic targets in *Plasmodium falciparum* [41, 42], *Leishmania mexicana* [53], *Trypanosoma brucei and Trypanosoma cruzi* [54, 55]. Chen et al. [56] reported that immunization with recombinant UCE induced protection against *Taenia pisiformis*. Our subsequent studies demonstrated that 14-3-3 protein and GAPDH could confer protection against coinfection of *E. tenella*, *E. acervulina* and *E. maxima* (unpublished data). Taken together, the five common immunodominant antigens could be selected as vaccine candidates against *Eimeria*.

We provided reference maps of sporozoite immunodominant proteins for *E. tenella*, *E. acervulina* and *E. maxima*. In some previous studies, sporozoites protein 2DE profiles of *Eimeria* have been reported [29, 57–60]. However, sporozoites immunodominant protein 2DE profiles were seldom reported. de Venevelles et al. analyzed the sporozoite 2DE map of *E. tenella* and detected approximately 50 immunodominant protein spots. However, they only identified a few of the immunodominant spots by mass spectrometry [29]. In this study, 50, 64 and 57 sporozoite immunodominant protein spots of *E. tenella*, *E. acervulina* and *E. maxima* were detected and identified as corresponding to 33 immunodominant antigens of *E. tenella*, 27 of *E. acervulina* and 25 of *E. maxima* respectively. Our results provided additional sporozoite immunodominant antigens and sporozoite immunodominant proteins reference maps for *E. tenella*, *E. acervulina* and *E. maxima*.

## MATERIALS AND METHODS

### Ethics statement

Animal experiments were conducted following the guidelines of the Animal Ethics Committee, Nanjing Agricultural University, China. All animal experiments were evaluated and approved by the Institutional Animal Care and Use Committee of Nanjing Agricultural University (approval number: 2012CB120750).

### Chickens and parasites

New-hatched Hy-Line layer chickens (commercial breed W-36) were reared in sterilized wire cages under coccidian-free conditions and provided daily with coccidiostat-free feed and water until the end of experiment. Oocysts of *E. tenella*, *E. acervulina* and *E. maxima* were propagated, harvested and sporulated using a previously described protocol [61], and then stored in 2% (w/v) potassium dichromate solution at 4°C no longer than 2 weeks. Purity of the parasites was determined by PCR based on the internal transcribed spacer-1 (ITS-1) as previously described [62, 63]. Sporozoites of the parasites were harvested from sporulated oocysts by *in vitro* excystation and purified over nylon wool and DE-52 cellulose columns [61].

### Antisera preparation

Three antisera were prepared by inoculation of two-week-old chickens with pure coccidian. Chickens were orally inoculated 6 times at 3-day intervals with 1 × 10^4^ sporulated oocysts of *E. tenella*, *E. acervulina* or *E. maxima* per chicken. Negative control birds were maintained under the same conditions and inoculated with distilled water. One week post the last inoculation, blood was collected from wing vein of the chickens. Subsequently, the sera were collected and determined by ELISA. A seventh even eighth dose would be given unless titers of the sera were beyond 1: 64. Sera were stored at −20°C for Western blot analysis. Meanwhile, serum was collected from uninfected chickens as negative control [21, 64].

### Two-dimensional electrophoresis (2DE)

Purified sporozoites of the three species were smashed in lysis buffer (7 M urea, 2 M thiourea, 4% CHAPS, 40 mM dithiothreitol (DTT), 0.2% Bio-Lyte 3–10 ampholytes and 1 mM PMSF) by ultrasonic in ice bath (200 W, work time 5 s, intervaltime10 s, 50 cycles). Soluble proteins were obtained after centrifugation for 1 min (15,000 rpm) at 4°C. Then the soluble proteins were treated with 2D clean up kit and quantified using PlusOne^™^ 2-D Quant Kit (Amersham Pharmacia) [65].

For 2DE, 400μg of sporozoite proteins were loaded onto analytical and preparative gels. The Ettan IPGphor Isoelectric Focusing System (GE Amersham) and pH 3–10 immobilized pH gradient (IPG) strips (13 cm, nonlinear; GE Healthcare) were used for isoelectric focusing (IEF). The IPG strips were rehydrated for 12 h in 250 μl of rehydration buffer containing the protein samples. IEF was performed in four steps: 30V for 12 h, 500 V for 1 h, 1000 V for 1 h, and 8000 V for 8 h. The gel strips were equilibrated for 15 min in equilibration buffer (50 mM Tris-HCl (pH 8.8), 6 M urea, 2% SDS, 30% glycerol, and 1% DTT). This step was repeated using the same buffer with 4% iodoacetamide in place of 1% DTT. The strips were then subjected to the second-dimensional electrophoresis after transfer onto 12.5% SDS-polyacrylamide gels. Electrophoresis was performed using the Hofer SE 600 system (GE Amersham). The 2DE was performed twice for each sample simultaneously, and one of obtained gels was used for immunoblot analysis, while the other one prepared for silver staining.

### Western blot and image analysis

Proteins in the 2DE gel were transferred electrophoretically onto a 0.45 μm pore size polyvinylidene fluoride (PVDF) membrane (GE Healthcare, USA) for 2 h at 100V using a TE62 Tank Transfer Unit system (GE Healthcare, USA). Membranes were then blocked in 5% skim milk in PBS containing 0.05% Tween 20 (PBST) for 1h at room temperature with gently swinging. And then was incubated with anti-*E. tenella*, anti-*E. acervulina* and anti-*E. maxima* sera (1:100) for 2 h at 37°C. The uninfected chicken serum was used to test another membrane as a negative control. After frequent washing with PBST, the membrane were incubated with secondary antibody of Goat anti-chicken IgG (1:2000, PTG Inc., USA) for 2 h at 37°C. Finally, 3, 3′-diaminobenzidine (DAB, Sigma) was added to visualize the immunodominant protein spots, according to the manufacturer's instructions.

Blots were scanned using TyphoonTMFLA 9500 (GE Amersham, USA). Through ImageMaster 2D Platinum (Version 5.0, GE Amersham, USA), the spots on the membranes were matched to their orthologs in 2DE gels stained using a modified silver staining methods compatible with subsequent mass spectrometric analysis [66].

### Two-dimensional gel excision, Tryptic Digestion, and Desalting

All the the immunodominant spots on the PVDF membranes were excised from 2D gels from the preparative gels. Subsequently, the protein spots were destained for 20 min in 30 mM potassium ferricyanide/100mM sodium thiosulfate (1:1 v/v) and washed with Milli-Q waters. The spots were incubated in 0.2 M NH_4_HCO_3_ for 20 min and then lyophilized. Each spot was digested overnight in 12.5 ng/μl trypsin in 25 mM NH_4_HCO_3_. The peptides were extracted three times with 60% acetonitrile (ACN)/0.1% trifluoroacetic acid (TFA). The extracts were pooled and dried completely by a vacuum centrifuge.

### MS analysis of protein spot and database searches

MS analysis of protein spot was performed by APT (Applied ProteinTechnology co. ltd, Shanghai, China). MS and MS/MS data for protein identification were obtained by using a MALDI-TOF-TOF instrument (5800 proteomics analyzer; Applied Biosystems). Instrument parameters were set using the 4000 Series Explorer software (Applied Biosystems). The MS spectra were recorded in reflector mode in a mass range from 800 to 4000 with a focus mass of 2000. MS was used a CalMix5 standard to calibrate the instrument (ABI 4700 Calibration Mixture). For one main MS spectrum 25 subspectra with 125 shots per subspectrum were accumulated using a random search pattern. For MS calibration, autolysis peaks of trypsin ([M+H]+842.5100 and 2,211.1046) were used as internal calibrates, and up to 10 of the most intense ion signals were selected as precursors for MS/MS acquisition, excluding the trypsin autolysis peaks and the matrix ion signals. In MS/MS positive ion mode, for one main MS spectrum 50 subspectra with 50 shots per subspectrum were accumulated using a random search pattern. Collision energy was 2 kV, collision gas was air, and default calibration was set by using the Glu1-Fibrino-peptide B ([M+H] + 1,570.6696) spotted onto Cal 7 positions of the MALDI target. Combined peptide mass fingerprinting PMF and MS/MS queries were performed by using the MASCOT search engine 2.2 (Matrix Science, Ltd.) embedded into GPS-Explorer Software 3.6 (Applied Biosystems) on the database of uniprot *Eimeria* or NCBI with the following parameter settings: 100 ppm mass accuracy, trypsin cleavage one missed cleavage allowed, carbamidomethylation set as fixed modification, oxidation of methionine was allowed as variable modification, MS/MS fragment tolerance was set to 0.4 Da. a GPS Explorer protein confidence index ≥ 95% were used for further manual validation.

## Abbreviations

*E. tenella*: *Eimeria tenella*
*E. acervulina*: *Eimeria acervulina*
*E. maxima*: *Eimeria maxima*
*E. mitis*: *Eimeria mitis*
2DE: two-dimensional electrophoresis
MALDI-TOF-MS/MS: Matrix-Assisted Laser Desorption/ Ionization Time of Flight Mass Spectrometry/ Mass Spectrometry
NCBI: The National Center for Biotechnology Information
spp.: species
EF2: Elongation factor 2
UCE: Ubiquitin-conjugating enzyme domain-containing protein
GAPDH: Glyceraldehyde-3-phosphate dehydrogenase
PBS: phosphate buffer saline
ITS-1: internal transcribed spacer-1
